# The affinities of the Late Triassic *Cryptovaranoides* and the age of crown squamates

**DOI:** 10.1098/rsos.230968

**Published:** 2023-10-11

**Authors:** Chase D. Brownstein, Tiago R. Simões, Michael W. Caldwell, Michael S. Y. Lee, Dalton L. Meyer, Simon G. Scarpetta

**Affiliations:** ^1^ Department of Ecology and Evolutionary Biology, Yale University, New Haven, CT 06511, USA; ^2^ Department of Earth and Planetary Sciences, Yale University, New Haven, CT 06511, USA; ^3^ Stamford Museum and Nature Center, Stamford, CT 06903, USA; ^4^ Department of Organismic and Evolutionary Biology & Museum of Comparative Zoology, Harvard University, Cambridge, MA 02138, USA; ^5^ Department of Ecology and Evolutionary Biology, Princeton University, Princeton, NJ 08544, USA; ^6^ Department of Biological Sciences, University of Alberta, Edmonton, Alberta, Canada; ^7^ Department of Earth and Atmospheric Sciences, University of Alberta, Edmonton, Alberta, Canada; ^8^ College of Science and Engineering, Flinders University, Adelaide 5001, Australia; ^9^ Earth Sciences Section, South Australian Museum, North Terrace, Adelaide 5000, Australia; ^10^ Museum of Vertebrate Zoology, Department of Integrative Biology, University of California, Berkeley, CA 94720, USA; ^11^ Department of Environmental Science, University of San Francisco, San Francisco, CA 94117, USA

**Keywords:** phylogenetics, divergence times, squamata, reptiles, Triassic, *Cryptovaranoides*

## Abstract

Most living reptile diversity is concentrated in Squamata (lizards, including snakes), which have poorly known origins in space and time. Recently, †*Cryptovaranoides microlanius* from the Late Triassic of the United Kingdom was described as the oldest crown squamate. If true, this result would push back the origin of all major lizard clades by 30–65 Myr and suggest that divergence times for reptile clades estimated using genomic and morphological data are grossly inaccurate. Here, we use computed tomography scans and expanded phylogenetic datasets to re-evaluate the phylogenetic affinities of †*Cryptovaranoides* and other putative early squamates. We robustly reject the crown squamate affinities of †*Cryptovaranoides*, and instead resolve †*Cryptovaranoides* as a potential member of the bird and crocodylian total clade, Archosauromorpha. Bayesian total evidence dating supports a Jurassic origin of crown squamates, not Triassic as recently suggested. We highlight how features traditionally linked to lepidosaurs are in fact widespread across Triassic reptiles. Our study reaffirms the importance of critically choosing and constructing morphological datasets and appropriate taxon sampling to test the phylogenetic affinities of problematic fossils and calibrate the Tree of Life.

## 1. Introduction

Modern amniote diversity is concentrated in three clades: mammals, birds and lizards, inclusive of snakes and amphisbaenians (Squamata). Of these, the more than 11 000 living species of squamates represent the most speciose modern tetrapod clade [1,2]. Living squamates are ecologically diverse and have colonized and diversified across all major landmasses except Antarctica [1–9]. Squamates and their sister clade, Rhynchocephalia, survived by the single species *Sphenodon punctatus*, comprise the Lepidosauria, which diverged from other reptiles between 245 and 270 Ma [1].

Recent additions and revisions to the early squamate and rhynchocephalian fossil records made possible by the use of new imaging and analytical tools have substantially improved our understanding of squamate origins and the rise of squamates to ecological dominance as rhynchocephalian diversity declined [6–10]. Reconstructing the divergence times of living squamate clades has been greatly aided by the reconciliation of major differences among phylogenies of squamates produced using morphological and molecular data [1,5,7,9,11]. These advances have consistently inferred a Triassic (approx. 252–202 Ma) age for the origins of the squamate and rhynchocephalian total clades, followed by the diversification of the major clades of crown squamates during the Jurassic (approx. 202–145 Ma) [5,7,11–15]. This was followed by the radiation of modern squamate families during the middle and Late Cretaceous [16,17] shortly after the Cretaceous Terrestrial Revolution [18], along with the Cretaceous invasion of aquatic environments by mosasaurian squamates [19].

**Figure 1.**
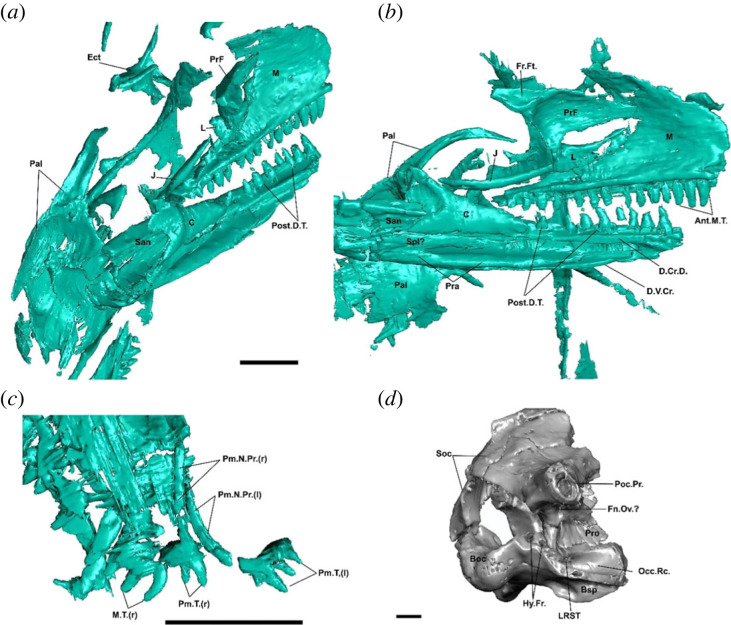
Cranial morphology of †*Cryptovaranoides microlanius* NHMUK PV R36822. (*a*) Skull and mandible of holotype in posteromedial view. (*b*) Skull and mandible of holotype in medial view. (*c*) Premaxillae and maxilla of holotype in dorsal view. (*d*) Isolated and mostly complete braincase (NHMUK PV 37377) in posterolateral view. Screen captures from computed tomography scan model in Whiteside *et al.* ([24]: electronic supplementary material). Ant.M.T., anterior maxillary teeth; Boc, basioccipital; Bsp, basiphenoid; C, coronoid; D.Cr.D., crista dorsalis of dentary; D.V.Cr., Ect, ectopterygoid; Fn.Ov., fenestra ovalis; Fr.Ft., frontal facet on prefrontal; Hy.Fr., hypoglossal foramina; J, jugal; L, lacrimal; LRST, lateral aperture of recessus scale tympanum; M, maxilla; M.T., maxillary teeth; Occ.Rc., occipital recess; Pal, palatine; Pm.N.Pr., premaxillary nasal process Pm.T., premaxillary teeth; Pra, prearticular; PrF, prefrontal; Pro, prootic; Poc.Pr., paraoccipital process; Post.D.T., posterior dentary teeth; San, surangular; Soc, supraoccipital; Spl, splenial; (r), right; (l); left. Scale bars = 2 mm.

**Figure 2.**
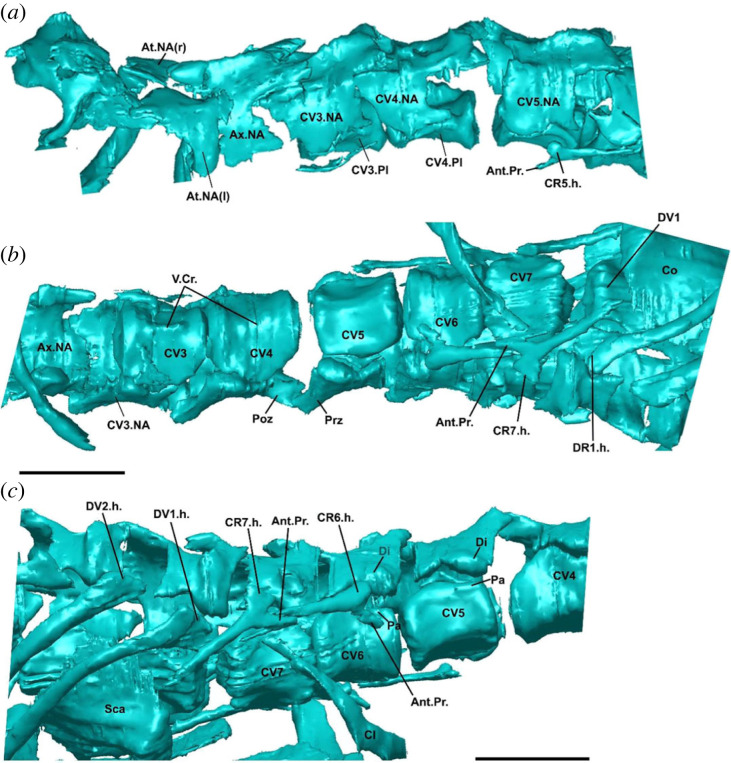
Postcranial morphology of †*Cryptovaranoides microlanius* NHMUK PV R36822. (*a*) Cervical vertebrae in left lateral view. (*b*) Cervical anteriormost dorsal vertebrae in ventral view. (*c*) Cervical and anteriormost dorsal vertebrae in right lateral view. Screen captures from computed tomography scan model in Whiteside *et al.* ([24]: electronic supplementary material). Ant.Pr., anterior process of cervical rib; At.NA., atlas neural arch; Ax.NA., axis neural arch; Cl, clavicle; CV(no.), cervical vertebra; CR(no.).h., cervical rib head (fused tuberculum + capitulum); CV(no.).NA., cervical vertebra neural arch; Di, diapophysis; DV(no.).h., dorsal rib head; Pa, parapophysis; Poz, postzygapophysis; Prz, prezygapophysis; Sca, scapula; V.cr., ventral crest; (r), right; (l); left; (no.), number of referred vertebra or rib. Scale bars = 2 mm.

**Figure 3.**
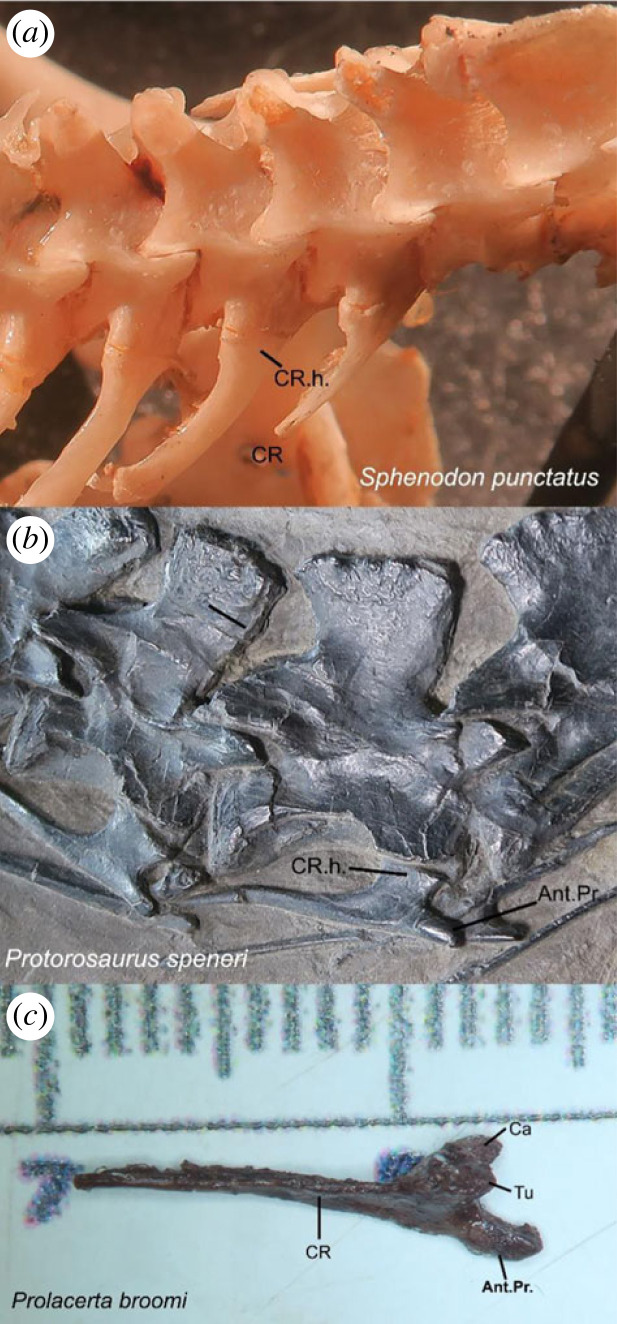
Comparative cervical rib morphology across reptiles. (*a*) Fused tuberculum and capitulum forming an expanded rib head in *Sphenodon punctatus* (common in sphenodontians and some early reptiles). In squamates the same pattern occurs, but the rib head is circular in cross section. (*b*) Same as in (*a*), but with a relatively elongate accessory anterior process in †*Protorosaurus speneri* (common among archosauromorphs). (*c*) Separate tuberculum and capitulum forming a double-headed cervical rib that also bears an elongate accessory anterior process in †*Prolacerta broomi* (variably occurring within archosauromorphs). Ant.Pr., anterior process of cervical rib; Ca, capitulum; CR, cervical rib; CR.h., cervical rib head; Tu, tuberculum.

**Figure 4.**
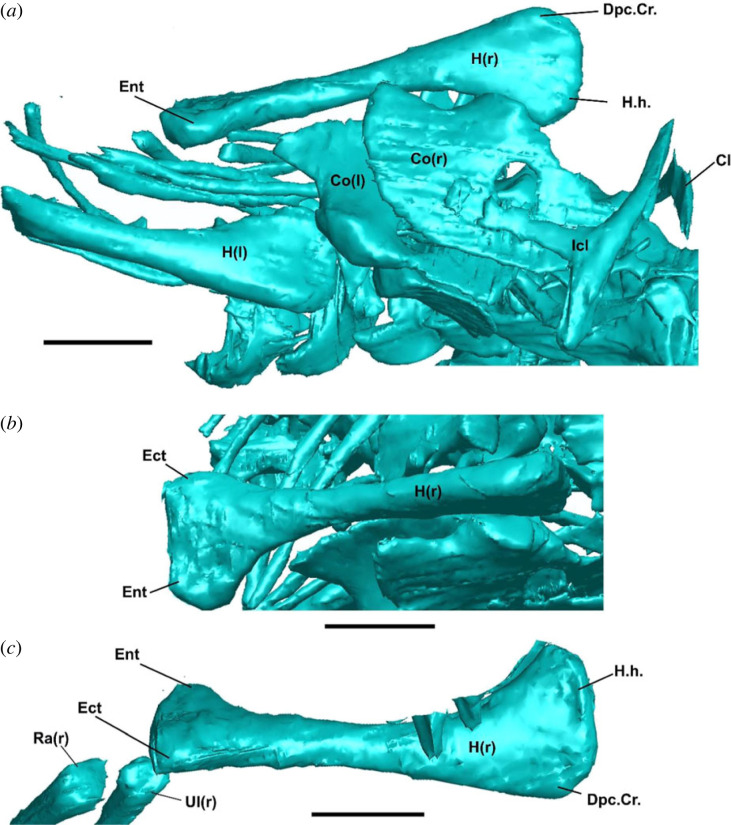
Postcranial morphology of †*Cryptovaranoides microlanius* NHMUK PV R36822. (*a*) Humeri and pectoral girdle in ventral view. (*b*) Right humerus in anterior view. (*c*) Right humerus in dorsal view. Cl, clavicle; Co, coracoid; Dpc.Cr., deltopectoral crest; Ect, ectopicondyle; Ent, entepicondyle; Icl, interclavicle; H, humerus; H.h., humeral head; (r), right; (l); left. Scale bars = 2 mm.

**Figure 5.**
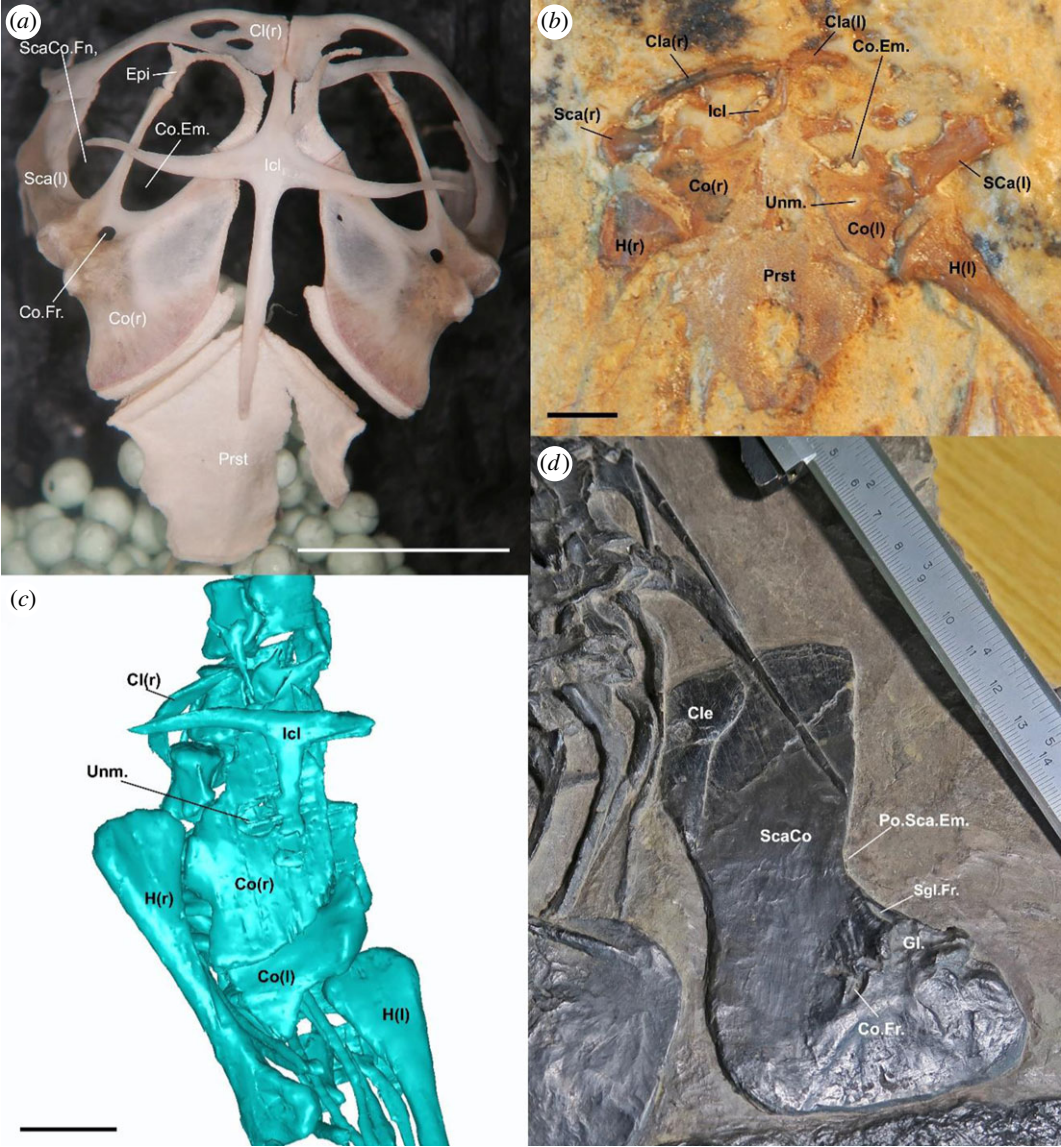
Comparative pectoral girdle morphology across reptiles. (*a*) Pectoral girdle of the extant lizard *Broadleysaurus major* (Gerrhosauridae: AMNH 173621). (*b*) Pectoral girdle of the fossil lizard †*Tijubina pontei*. (*c*) Pectoral girdle of the holotype of †*Cryptovaranoides microlanius* NHMUK PV R36822 (screen capture of computed tomography data provided in Whiteside *et al*., [24]). (*d*) Pectoral girdle of the protorosaur †*Protorosaurus speneri* (WMsN P 47361). Cl, clavicle; Cle, cleithrum; Co, coracoid portion of scapulacoracoid; Co.Em., coracoid emargination; Co.Fr., coracoid foramen; Epi, epicoracoid; Gl., Glenoid; Icl, interclavicle; H, humerus; Po.Sca.Em., posterior scapular emargination; Prst, presternum; Sca, scapula portion of scapulacoracoid; ScaCo, scapulacoracoid; ScaCo.Fn., scapulacoracoid fenestra; Sgl.Fr., supraglenoid foramen; Unm., unmineralized region; (r), right; (l); left. Scale bars = 10 mm (*a*) and 2 mm (*b,c*).

The Triassic-Jurassic fissure fill deposits of Gloucestershire and Somerset in the United Kingdom are known for producing an important fossil record of early lepidosaurs [20–23]. Recently, Whiteside *et al*. [24] described a surprising addition to this fauna, †*Cryptovaranoides microlanius* (figures 1–5), which they hypothesized as having phylogenetic affinities to Anguimorpha (monitors, Gila monsters, slow worms, alligator lizards, etc.), thus being deeply nested within the squamate crown—some analyses even placed †*C. microlanius* as the sister taxon to the living genus *Xenosaurus* [24]. Anguimorphs are otherwise universally inferred to have diverged from other squamates between the Early Jurassic and the Late Cretaceous based on total evidence dating (morphological and molecular) analyses [7,9,10] or genomic timetrees [5,14,25,26]. If †*C. microlanius* is an anguimorph squamate as Whiteside *et al*. [24] hypothesized, this species would push back the origins of major squamate crown clades by 30 to as much as 65 Myr. This result would suggest that all previous time-calibrated phylogenies using genomic or total evidence dating have grossly underestimated the age of crown squamates, crown lepidosaurs and potentially even crown reptiles, the oldest of which are middle to late Permian in age [27]. Such old age estimates would further imply that essentially all analyses dating the reptile Tree of Life failed to recover reasonable divergence times even for clades with (apparently) well-sampled fossil records and for which genome-scale sequence data were available. Given the potential impact of †*C. microlanius* on the current timescale of vertebrate evolution, the affinities of this species to crown Squamata must be rigorously tested.

Here, we re-evaluate the description of †*C. microlanius* using the computed tomography (CT) scan data available online [20] for the holotype and some referred specimens of †*C. microlanius*, as well as a wealth of comparative CT data and personal observations. We re-examined the original diagnosis relative to the referred specimens and holotype and re-assessed the primary homology concepts applied to create character scorings from the original study. Our detailed re-assessment of †*C. microlanius* reveals that the concept of the taxon was based on multiple fossil specimens that were not discovered in association with each other, save for the elements in the holotype block, and that there is little to no anatomical justification for the referral of these morphologically disparate skeletal remains to the same species (see details of the original referrals in Whiteside *et al*. [24]). Further, our close inspection of the CT data of the holotype reveals several errors in its original anatomical description, especially in the postcranium, which collectively reveal the absence of several anatomical features used to link this taxon to squamates more broadly, and anguimorphs more specifically.

We tested the phylogenetic affinities of †*C. microlanius* and divergence times for the major groups of squamates using three radically different phylogenetic matrices (updated to include several recently published early lepidosaurs and early squamates) under various optimality criteria. We find no support for the placement of †*C. microlanius* within Anguimorpha or even crown Squamata, with a broader reptile dataset suggesting that it is in fact an archosauromorph reptile. Divergence time estimates support previous estimates for a Mid-Late Jurassic origin of anguimorphs, highlighting how misinterpretations of the fossil record can highly impact our understanding of the origin of major branches of the Tree of Life.

## 2. Material and methods

### 2.1. Phylogenetic datasets

The data matrices used by Whiteside *et al*. [24] to assess the phylogenetic affinities of †*C. microlanius* are not ideal, as nearly all the non-lepidosaur species included in the original versions of those matrices were excluded. This approach potentially compromises the ability of the matrix to provide a strong test of whether †*C. microlanius* falls outside crown Squamata. Therefore, we included †*C. microlanius* in the largest available dataset to infer relationships among the major groups of reptiles [28], as it extensively samples early amniotes, the early radiation of the major groups of reptiles, and the two major reptile crown groups: lepidosauromorphs and archosauromorphs (‘dataset 1’ herein). Lepidosauromorphs were sampled in this dataset according to recent advances and increasing congruence among morphological and molecular hypotheses concerning the early part of the squamate tree of life (see Simões & Pyron [1] for a detailed review on the topic). We suggest that dataset 1 herein (see below) should be used to test higher-level reptile phylogenetic questions (e.g. composition of the Lepidosauromorpha or other major groups of reptiles), whereas lepidosaur-focused datasets (e.g. datasets 2 and 3 herein, see below) can be used to address phylogenetic questions within squamates and other lepidosaurs. Besides the addition of †*C. microlanius*, we further expanded the taxonomic sampling of dataset 1 by adding three taxa with relatively unstable relationships, but which have historically been linked to Lepidosauria: †*Fraxinisaura rozynekae* from the Middle Triassic of Germany [29], and †*Palaeagama vielhaueri* [30] and †*Paliguana whitei* [31] from the Late Permian-Early Triassic of South Africa. We also included new data provided by recently published CT scans of †*Pali. whitei* [32] (see further details in the electronic supplementary material). Importantly, the data collected on these species were based on personal observations of their holotypes (and only available specimens for the first two species). In total, dataset 1 comprises 129 taxa and 348 characters.

Subsequently, we tested the phylogenetic placements of †*C. microlanius* in datasets focused on lepidosaurs and squamates [6,7] using several updates on two different datasets produced over recent years (e.g. [9,10,33]). One of these datasets (dataset 2 herein: Simões *et al*. [7] and its subsequent expansions, (e.g. [32,33])) is the same as the one used by Whiteside *et al*. [24] for their initial phylogenetic assessment of †*C. microlanius*, allowing for direct comparisons to the results presented in that study [24]. To this dataset, we made substantial updates by merging various changes and additions from several recent studies [10,32–38] into a single version. Further, we added new taxa, revised recently added characters, and corrected previous data scores (see details in the electronic supplementary material). After critically evaluating all scorings of †*C. microlanius* provided by Whiteside *et al*. [24] in dataset 2, we made a substantial number of corrections (taxon score changes are illustrated in the electronic supplementary material, Data). We based our scorings solely on the holotype of this species, as the other materials referred to this species vary considerably in size and morphology and potentially represent different taxa (table 1), or at least different ontogenetic stages (Whiteside *et al*. [24] suggested the latter hypothesis). In brief, nearly all squamate and anguimorph synapomorphies that Whiteside *et al*. [24] proposed were shared by †*C. microlanius* are either ambiguously present in the holotype or referred material, or of questionable homology in †*C. microlanius* and squamates, or simply are not preserved (table 1).

**Table 1.** Referred specimen attribution to holotype of *Cryptovaranoides microlanius*.

specimen	element(s)	differences from holotype	figured by [24]	conclusion
NHMUK PV R36822	partial skull, mandible, cervical, dorsals and pectoral region	—	figures 1,5,6,7	—
NHMUK PV R36999	left maxilla	non substantial	figure 3	referrable
NHMUK PV R37279	right maxilla	specimen smaller than NHMUK PV R36999, but with larger tooth count, so it cannot be related to ontogeny. It could potentially reflect population differences. Facial process broken and not comparable. Similar dental morphology	figure 4	ambiguous
NHMUK PV R37280	fragment of right maxilla	tooth crowns shape is different from holotype and carinae are absent on the holotype and referred NHMUK PV R36999	figure 3	not referrable
NHMUK PV R37282	fragment of left dentary	tooth crowns shape is different from holotype and carinae are absent on the holotype and referred NHMUK PV R36999	figure 3	not referrable
NHMUK PV R37001	left dentary	tooth morphology is very similar to holotype. However, symphysial region may be different in holotype (currently much shallower in NHMUK PV R36822, although it may not be fully preserved). The ventral margin of the dentary is very robust here whereas is it is quite thin in the holotype	figure 3	ambiguous
NHMUK PV R37281	right dentary	unknown	not figured	ambiguous
NHMUK PV R37273	left coronoid	non substantial	figure 7	referrable
NHMUK PV R37274	left frontal	no comparable element in holotype	figure 7	ambiguous
NHMUK PV R37604	left quadrate	non substantial	figure 6	referrable
NHMUK PV R37377	braincase	this isolated braincase shares no overlapping features with the holotype. The isolated basioccipital in figure 1*d* is by the authors admission, not from the holotype individual, and is two to three times too large for the holotype skull and basisphenoid	figure 4	not referrable
NHMUK PV R37378	premaxilla	isolated specimen. Dental crown apices are broken and so are not comparable to holotype. Incisive process indicated here by the authors is not visible in holotype premaxilla	figure 4	ambiguous
NHMUK PV R37275	sacral vertebrae	no comparable element in holotype	figure 7	ambiguous
NHMUK PV R37276	cervical vertebrae	unknown	not figured	ambiguous
NHMUK PV R37277	dorsal vertebrae	unknown	not figured	ambiguous

Finally, dataset 3 is a recent update of Gauthier *et al*. [6] by Brownstein *et al*. [9], that samples the major living squamate family-level clades and a majority of Mesozoic squamates known from relatively complete skulls and skeletons. Datasets 2 and 3 are very different in their logic of character construction (see discussions in [8]), taxonomic sampling (higher proportion of fossils and other lepidosaurs versus squamates only), and data type (combined evidence versus morphology only). Our intent with using two vastly different datasets is to provide a thorough testing of the original hypothesis of †*C. microlanius* being deeply nested within squamates, regardless of the results presented by dataset 1.

### 2.2. Tree search procedures

#### 2.2.1. Maximum parsimony

All maximum-parsimony (MP) analyses were conducted in T.N.T. v. 1.5 [39] which allows a better sampling of all possible local optima of most parsimonious trees (MPTs) for datasets with a large taxon sample. Searches were conducted using a combination of multiple New Technology Search algorithms, namely successive rounds of Ratchet (1000 iterations), Sectorial Search (1000 rounds) and Tree Fusing (1000 rounds) upon 1000 initial trees obtained with random addition sequences. This procedure provides shorter MPTs in comparison with other MP searches implemented with PAUP* v.4.0 [40] by previous studies [41].

#### 2.2.2. Bayesian inference analyses

Bayesian analysis of the morphological dataset was performed in MrBayes v. 3.2.7a [42] using the Odyssey HPC cluster at Harvard University.

We used the Mkv + gamma substitution model [43] (Mk with ascertainment bias correction) for the morphological data of datasets 1 and 2. The molecular partition of the combined evidence dataset (dataset 2) was analysed under the GTR + gamma model and subdivided into four partitions, following the previous partitioning and model test analyses for this dataset [11,35].

Dataset 1 was run for 30 million generations, under four independent runs with four chains each, temperature = 0.007, 25% burn-in, and sample frequency at every 1000 generations. In dataset 2, the morphology only analysis was run with 40 million generations, four runs with six chains, temperature = 0.01, 25% burn-in, and sample frequency at every 1000 generations. The combined evidence analysis of dataset 2 used similar parameters but ran over 50 million generations.

Stationarity was assessed using standard measures, such as average standard deviation of split frequencies (ASDSF < 0.05) and potential scale reduction factors (PSRF ≈ 1 for all parameters). Effective sample size values were assessed using Tracer v.1.6 [44], reaching greater than 200 for all parameters. Our reported summary trees were calculated with standard output tree procedures available in MrBayes: the majority rule consensus tree and the maximum compatible tree (MCT).

### 2.3. Divergence time estimates

Divergence times were calculated using relaxed clock Bayesian inference analyses of the morphological dataset for dataset 1 and the combined molecular and morphological data for dataset 2. We implemented total-evidence-dating (TED) using the fossilized birth-death tree model, under relaxed clock models in MrBayes v. 3.2.7a [42].

#### 2.3.1. Divergence times for dataset 1

The tree model and its calibration priors follow the previous analyses of this dataset in [28]. Namely, the sampling strategy was set to the ‘random’ sampling strategy which assumes that all taxa are sampled randomly. Further, a recent study showed that accounting for sampled ancestors in conditions where they are highly unlikely to occur (extremely sparse taxon sampling across time and space) induce a deep root attraction problem and unreasonably older divergence times [11]. Thus, we forced all fossils to be tips only (no sampled ancestor model, *ρ* = 1). The age of the root also follows [28], sampled from an offset exponential distribution with a hard bound for the minimum age (based on the minimum age for the oldest amniote fossils at 315 Ma; [28]) and a soft maximum age, with the mean of the exponential distribution based on the upper range of previous molecular clock estimates for this node (330 Ma; [28]). The range of the stratigraphic occurrence of the fossils used for tip-dating here was used to inform the uniform prior distributions on the age of those same fossil tips (thus allowing for uncertainty on the age of the fossils). Given the very few changes on this dataset, we followed the same clock models as provided in the previous analyses of this dataset in [28]. Specifically, an informative prior to the base of the clock rate based on the previous non-clock analysis: −3.3242 in log scale and a wide standard deviation (1.0). We employed the TK02 autocorrelated relaxed clock model [45], which is the best fit to this dataset [28].

#### 2.3.2. Divergence times for dataset 2

Sampling strategy among extant taxa was set to ‘diversity’, which is more appropriate when sampling maximizes extant diversity (as performed herein) and fossils are assumed to be sampled randomly [42,46]. Accounting for diversity, sampling impacts tree priors [47], improving divergence time precision and accuracy as with dataset 1, we used a no sampled ancestor model (*ρ* = 1) where all fossils are considered to be tips only.

The age of the root (node representing the most recent common ancestor of †*Youngina* and crown reptiles) was sampled from an offset exponential distribution with a hard bound for the minimum age (based on the minimum age for †*Youngina*, at 255 Ma) and a soft maximum age, with the mean of the exponential distribution based on a recent TED analysis [28] for this node (280 Ma). This provides a relatively low but non-zero probability for sampling ages older than the maximum age for the root.

The vast majority of our calibrations were based on tip-dating, which accounts for the uncertainty in the placement of extinct taxa and avoids the issue of constraining priors on taxon relationships when implementing bound estimates for node-based age calibrations [48,49]. The range of the stratigraphic occurrence of the fossils used for tip-dating here were used to inform the uniform prior distributions on the age of those same fossil tips. However, in clades for which we lacked some of the oldest known fossils in our analysis, and for which there is overwhelming support in the literature (and in all our other analyses) regarding their monophyly, and for which the age of the oldest known fossil is well-established, we employed node age calibrations with a soft minimum age. These clades and calibrations are as follows: Serpentes: based on †*Eophis underwoodi* (Bathonian, Middle Jurassic—UK) [50] → 168.3–166.1 Ma (166.1,168.3) [51]; Sphenodontia: based on cf. †*Diphydontosaurus* (Ladinian, Middle Triassic—Germany) [12]→ 241.5–237 Ma (237 241.5) [51].

We provided an informative prior to the base of the clock rate based on the previous non-clock analysis: the median value for tree height in substitutions per character from posterior trees divided by the age of the tree based on the median of the distribution for the root prior: 13.1/280 = 0.0464, in natural log scale = −3.07, and a wide standard deviation (1.0). We employed the uncorrelated independent gamma rate clock model [52] as in previous iterations of this dataset. The prior on the variance of clock rates (informing individual branch rates) was linked across molecular partitions but unlinked between molecular and morphological partitions, as in previous iterations of this dataset [7].

## 3. Results

### 3.1. Systematic palaeontology

Reptilia Laurenti, 1768

†*Cryptovaranoides* Whiteside *et al*. [24].

†*Cryptovaranoides microlanius* Whiteside *et al*. [24].

#### 3.1.1. Holotype

NHMUK PV R36822, a partially articulated skeleton of a single, small reptile preserved in matrix. The presence of a large, isolated interclavicle in the block including the holotype demonstrates that additional reptiles (cf. *Clevosaurus* sp.; Whiteside *et al*. [24]) are represented in the †*Cryptovaranoides* type block. This observation motivated us to critically re-evaluate the referral of additional material described by Whiteside *et al*. [24] to †*Cryptovaranoides* (electronic supplementary material).

#### 3.1.2. Revised diagnosis

Pterygoid anterior process considerably longer than posterior process (figure 1*a*); coronoid bone that is 40% of the anteroposterior length of the dentary and forms a low, gently rising coronoid process (figure 1*b*); surangular as long as dentary (figure 1*b,c*); absence of incipient or developed rugosities and osteoderms on cranial bones (figure 1*d*); seven cervical vertebrae (figure 1*c*).

#### 3.1.3. Comments

The diagnosis of †*C. microlanius* provided by Whiteside *et al*. [24] cited several characters that are in fact widely distributed and present together across squamates and other reptiles, and therefore cannot substantially distinguish this taxon from others [3,6,7]. Furthermore, some of the suggested diagnostic features are unobservable or not preserved in the holotype and referred specimens (electronic supplementary material). The revised diagnosis above is based on our re-examination of the holotype specimen CT data; a revised description of this taxon is provided in the electronic supplementary material, accompanied by a checklist of all re-interpreted characters and comparative figures displaying several characters not illustrated by Whiteside *et al*. [20].

### 3.2. Anatomical and taxonomic re-interpretations of †*Cryptovaranoides microlanius*

In this section, we review the anatomy of †*C. microlanius* and note issues with the description presented in Whiteside *et al*. [24] based on CT scan data of the type specimen provided in its original publication. For the sake of clarity, we have restricted this section to detailing points of contention with that study rather than a comprehensive re-description of the anatomy of this taxon. Furthermore, some of the materials referred to †*C. microlanius* were found in isolation and lack diagnostic features that would make it referable to the same species of the holotype. Besides the anatomical reinterpretations below, we also provide a list of all specimens referred to †*C. microlanius* by Whiteside *et al*. [24], noting if there is any evidence to justify its referral to the holotype (table 1). Based on this evidence and our new interpretations, we have made several corrections to the scores for †*C. microlanius* in the data matrices used to infer its phylogenetic position.

#### 3.2.1. External skull

*Fusion of the premaxillae.* Whiteside *et al*. [24] described the premaxillae of †*C. microlanius* as fused, with a median tooth placed centrally. The presence of fused premaxillae is historically considered to be a squamate synapomorphy [3,6] and more recently, a synapomorphy of Unidentata—the group including all squamates except Gekkota and Dibamidae—independently re-evolving within some geckos [8,9]. As such, the presence of fused premaxillae bearing a median tooth in †*C. microlanius* would strongly support a squamate identity for this species. However, our re-evaluation of the CT scan data available for the holotype shows that the premaxillae are clearly unfused, and no median tooth is identifiable (figure 1*c*). The isolated premaxilla (NHMUK PV R37378) referred to †*C. microlanius* by Whiteside *et al*. [24] (figure 4*b*,*c*) cannot be directly compared to the holotype specimen because all the teeth of the referred premaxilla are broken. Furthermore, although this referred specimen does appear to show some degree of fusion of the premaxillae near the tooth row margin, the premaxillae are separated throughout most of their extension, and the apparent fusion could be an artefact of preservation (e.g. suture infilling by surrounding sedimentary matrix).

*Lacrimal arches dorsally over lacrimal duct and floors lacrimal duct with medial process posteriorly.* This feature was described as present in †*Cryptovaranoides microlanius* by [24], who argued that it united †*C. microlanius* with Anguimorpha. This feature is unobservable and cannot be scored for this taxon, as this region of the skull is disarticulated, and the lacrimal region is fragmented (figure 1*a*,*b*).

*Absence of a jugal posterior process.* This widely cited character is related to the partial or complete loss of the lower temporal bar ancestrally in squamates, although it also appears in some stem lepidosaurs and rhynchocephalians [3,6,7,29,33]. Whiteside *et al*. [24] suggested that the absence of a jugal posterior process was a pan-squamate synapomorphy that is also present in †*C. microlanius*. However, as noted, this condition is also found elsewhere in lepidosaurs and in many other neodiapsids, such as kuehneosaurids, sauropterygians and ichthyosaurs [7,28,53–55]. As such, this feature does not necessarily unite †*C. microlanius* with Squamata over other clades of neodiapsids. Furthermore, the posterior region of the jugal is broken on the holotype of †*C. microlanius* (figure 1*a*,*b*), and so it is possible that a posteroventral process was present but not preserved.

*Peg-in-notch articulation with rod-shaped squamosal.* This feature was cited as a potential squamate synapomorphy of †*C. microlanius* [24]. However, the postorbital and temporal regions of the skull are largely disarticulated in the holotype specimen of this taxon (figure 1*a–c*). The long, thin bone identified as the squamosal by Whiteside *et al*. [24] is oriented perpendicular to the tooth-bearing bones of the right side of the skull and is not articulated with the quadrate (figure 1). We tentatively agree that the squamosal and quadrate articulated over a limited region at the posterior end of the former, cf. Whiteside *et al*. [24], but it is currently interpreted as an ambiguous trait for †*C. microlanius*.

*Quadratojugal not present as separate element.* This feature was also listed as a squamate synapomorphy of †*C. microlanius* by Whiteside *et al*. [24]. We disagree with that interpretation, as (i) the quadratojugal cannot be identified as separate, fused or absent owing to the preservation of the holotype (an ontogenetic series would be ideally required), and (ii) the lateral margin of the quadrate, onto which the quadratojugal would attach or fuse, is broken. Therefore, the condition of the quadratojugal should be treated as missing data.

*Frontal underlaps parietal laterally on frontoparietal suture.* This articulation was described as an anguimorph synapomorphy that is present in †*C. microlanius* based on the inferred articulation of the frontals with the parietals [24]. However, the frontals are not preserved in the holotype (figure 1*a*,*b*), and the referred frontals were found in isolation and cannot be anatomically connected to any of the other preserved elements in the skull without ambiguity (table 1). Therefore, no frontal characters can be coded for †*C. microlanius*. We also note that lap sutures between the frontals and parietals vary considerably in squamates [3,6]. Because the frontals and parietals are not articulated in the holotype of †*C. microlanius*, the mode of frontal underlap cannot be assessed.

#### 3.2.2. Braincase

*Subdivision of metotic fissure by the crista tuberalis into vagus* (*jugular*) *foramen and recessus scala tympani.* Whiteside *et al*. [24] described this feature as a squamate synapomorphy of †*C. microlanius*. Whiteside *et al*. [24] acknowledged that the exit for the vagus nerve (vagus or jugular foramen) is not preserved in the braincase of †*C. microlanius*—see also figure 1*d*. Despite this, Whiteside *et al*. [24] still inferred the presence of this exit foramen without further justification. Without a direct observation of the location of the vagus foramen, it is not possible to tell whether nerve X had its own separate exit (as in squamates; [6,7,9]) or if it shared the exit with nerve IX through the lateral aperture for the recessus scala tympani within the occipital recess (the metotic fenestra of other reptiles). Therefore, this character must be treated as missing data.

*Enclosed vidian canal exiting anteriorly at base of each basipterygoid process.* This braincase feature was described as a squamate synapomorphy of †*C. microlanius* [24]. However, the opening identified could also be a blind recess. As Whiteside *et al*. [24] noted, cross-sections showing the canal extending through the bone appear to be showing discontinuous lacunae rather than a single canal, and since the braincase is well-preserved in three dimensions, this is unlikely to be an artefact of crushing.

*Fusion of exoccipitals and opisthotics forming an otoccipital.* This feature was referred to as a squamate synapomorphy of †*C. microlanius* [24]. Although we verified the presence of this feature (figure 1*d*), we note that braincase fusion is quite variable within Squamata [6] and other reptiles.

#### 3.2.3. Palate

*Septomaxilla probably contacts dorsal surface of palatal shelf of maxilla* (*septomaxillary facet on maxilla*)*.* This feature was described by Whiteside *et al*. [24] as a squamate synapomorphy present in †*C. microlanius*. However, these are completely disarticulated and the septomaxilla is not preserved (figure 1*a*–*c*).

*Long ventral longitudinal ridges converging toward midline of vomer.* This feature was described by Whiteside *et al*. [24] as an anguimorph synapomorphy present in †*C. microlanius*. The vomers of †*C. microlanius* are large, flattened, and subrectangular, and are more similar to the vomers of non-squamate lepidosaurs (e.g. †*Gephyrosaurus bridensis* [56]) than to squamates [6,9]. The anteroposteriorly trending ridges on the vomers of †*C. microlanius* are also very different from the crest-like ridges of anguimorphs [6], such as *Pseudopus apodus* [57] and *Elgaria* spp. [58,59], where these features appear as the apices of developed wings of bone. Further, new information on the palatal anatomy of early diverging crown squamates shows that ventral ridges on the vomer are found outside Anguimorpha, including in members of the pan-scincoid clade †Paramacellodidae [9].

*Prominent choanal fossa on palatine.* This feature was described as an unambiguous synapomorphy of Squamata present in †*C. microlanius* [24]. However, the construction of the choanal fossa is highly variable within lepidosaurs and is related to the construction of a secondary palate in some squamate clades [3,6,7,9]. The choanal fossa of †*C. microlanius* is deep and anteroposteriorly restricted, matching the condition in gekkotans [6,60,61] and the stem squamates †*Megachirella watchleri* [7], †*Bellairsia gracilis* [10] and †*Oculudentavis* spp. [62].

Stem and crown group members of the other major squamate clades display deeper and more posteriorly extensive choanal fossae [6,9,63], whereas the choanal fossa is nearly absent in non-squamate lepidosaurs such as †*Gephyrosaurus bridensis* [56], †*Marmoretta oxoniensis* [34], †*Taytalura alcoberi* [33], and sphenodontians—e.g. *Sphenodon punctatus* [6] and †*Navajosphenodon sani* [64]. Intriguingly, the choanal fossa of †*C. microlanius* is mediolaterally restricted so that it barely fills half of the mediolateral length of the anterior margin of the palatine [24]. This condition, though rare or absent in living lizards [6] or Mesozoic species with deep choanal fossae [9,65] is present in some archosauromorphs, including †*Tanystropheus hydroides* [48] and †*Macrocnemus bassanii* [66].

*Short overlap in quadrate-pterygoid contact and the absence of the pterygoid process on the quadrate.* This feature was described as a synapomorphy of Squamata found in †*C. microlanius* by Whiteside *et al*. [24]. However, the quadrate bone is broken medially where its pterygoid process would have been located. Furthermore, the pterygoid and quadrate are entirely disarticulated, and so the extent of the overlap of these two bones and the presence of a pterygoid process on the quadrate cannot be determined.

#### 3.2.4. Mandible

*Angular does not extend posteriorly to reach articular condyle.* Although the angular was suggested to terminate before the mandible articular condyle in †*C. microlanius* as in the squamate total clade [24], the posterior portion of the angular is not observable in the holotype or any specimen referred to this species (figure 1*a*,*b*).

**Figure 6.**
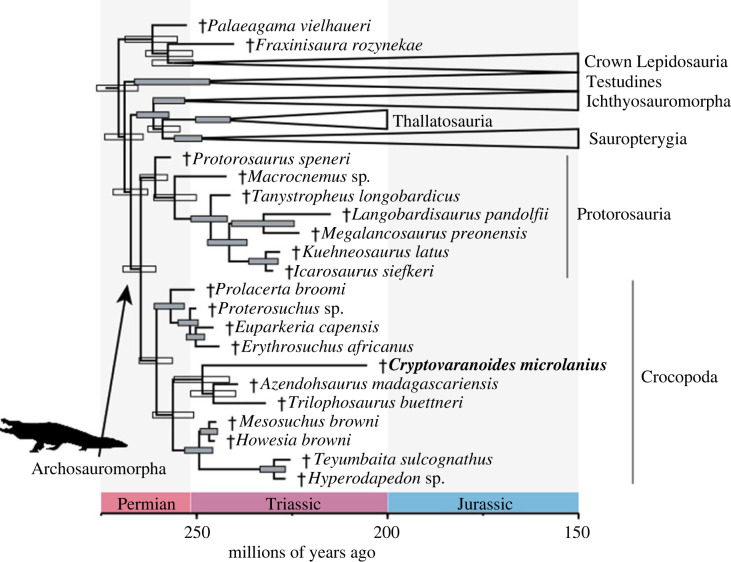
Phylogenetic relationships of †*Cryptovaranoides microlanius* among reptiles using morphological data in dataset 1 (summarized tree). Maximum compatible tree (MCT) inferred using tip-dated Bayesian analysis (after rogue taxon *Paliguana* excluded). Node bars indicate 95% highest posterior density intervals (HPDs), and grey bars indicate posterior support at nodes greater than 0.80. Daggers (†) indicate extinct species. For full trees, see the electronic supplementary material, figures S4–S9.

*Articulars and prearticulars medial process present.* This feature was scored as present in †*C. microlanius* by Whiteside *et al*. [24]. However, our inspection of the CT scan data shows no sign of a medial process (figure 1*a*,*c*). The absence of a medial process of the articular and prearticular can also be noticed in [24]: figure 6*g*,*h*.

#### 3.2.5. Postcranium

*Atlas pleurocentrum fused to axis pleurocentrum.* The fusion of these elements cannot be assessed because their pleurocentra are not preserved in the holotype. Only the neural arches and neural spine of the atlas and axis are preserved, and their intercentra are missing from the holotype (figure 2*a*,*b*).

*Cervical ribs double-headed.* Whiteside *et al*. [24] described double-headed ribs in †*C. microlanius*, acknowledging this state was unusual for a squamate. However, inspection of the CT data indicates that the cervical ribs of †*C. microlanius* are in fact single-headed and possess an expanded endpoint for articulation with the vertebral centra (figure 2*b*,*c*). This differs from the condition observed in all known squamates and resembles the rib morphology observed in other reptile clades, including protorosaurs such as †*Protorosaurus speneri* (figure 3), †*Tanystropheus hydroides* [67] and †*Macrocnemus bassanii* [66].

*Cervical ribs with an anteriorly oriented process* (*new observation*)*.* What was originally interpreted as the second rib head in †*C. microlanius* by Whiteside *et al*. [24] is reinterpreted as the anteriorly directed accessory process (figure 2*b*,*c*—Ant.Pr.) commonly observed on the cervical ribs of several archosauromorphs, including †*Protorosaurus speneri* (figure 3*b*), †*Prolacerta broomi* (figure 3*c*), †*Mesosuchus browni* [68], †*Azendhosaurus madagascariensis* [49] and several archosauriforms, such as proterosuchids [69] and †*Euparkeria capensis* [70]

*Cervical and dorsal vertebral intercentra present.* Based on the number of preserved, articulated vertebrae, the presence of intercentra on the trunk vertebrae was described by Whiteside *et al*. [24] as a squamate feature present in †*C. microlanius*. However, the presence of intercentra was based on the presence of a single isolated bone fragment, suggested as a displaced intercentrum. Upon inspection of the cervical region using CT scan data (figure 2*c*), we observed that cervical centra are in close articulation without any evidence for intercentra or articulatory facets for them, which should be clearly visible in this particularly well-preserved specimen, as they are in extant squamates (T. R. Simões 2023, personal observation and [6]). Therefore, we consider that the cervical intercentra are absent in †*C. microlanius*.

*Cervical vertebrae midventral crest absent.* A midventral crest or keel on each caudal centrum was scored as absent in †*C. microlanius* by Whiteside *et al*. [24]. However, our inspection of the CT scan data shows the unambiguous presence of a midventral crest on the cervical vertebrae of this species (figure 2*b*).

*Anterior dorsal vertebrae, diapophysis fuses to parapophysis.* Whiteside *et al*. [24] suggested that these processes are fused in the anterior dorsals of †*C. microlanius*. However, the few preserved dorsals have unfused neural arches and pleurocentra (figure 2*c*), thus logically having their diapophyses (located in the neural arches) and parapophyses (located on the pleurocentrum) also unfused. Given the juvenile condition of the holotype specimen (e.g. which is verified by the unfused neural arches and pleurocentra: electronic supplementary material, figure S3), it is possible that later during ontogeny those elements could have been fused together, forming a synapophysis. However, there is no evidence to support this given the material available. Secondly, even if synapophyses occur later in the ontogeny of †*C. microlanius*, these are observed across several groups of reptiles, including all other non-squamate lepidosaurs [7,28,68,71,72], and thus are not exclusive to squamates.

*Zygosphene-zygantra in dorsal vertebrae.* Incipient zygosphene-zygantra articulations were mentioned to be present in a set of vertebrae present in the block containing the holotype but separate from the holotype specimen of †*C. microlanius* [24]. However: (i) these structures were mentioned but not illustrated the original description; (ii) these vertebrae were found in isolation and cannot be anatomically linked to the holotype, making them non-referable to †*C. microlanius* (table 1); and (iii) even among the best-preserved vertebrae in the holotype, there is no evidence of any accessory vertebral articulatory facets, such as zygosphene-zygantra (figure 2). We consider the latter sufficient evidence to consider this feature absent in †*C. microlanius*.

*Anterior and posterior coracoid foramina present.* These foramina were originally interpreted to penetrate the coracoid of the holotype specimen of †*C. microlanius* by [24]. We note that this feature as observed in †*C. microlanius* (figures 4*a* and 5*c*) is quite different from the coracoid emarginations (or ‘foramina’ as labelled by [24]) observed in squamates (figure 5*a*). We reinterpret them as an instance of incomplete mineralization of the central region of the coracoid, which is common among juvenile reptiles (T. R. Simões 2023, personal observation)—e.g. a similar mode of preservation occurs in the coracoid of the Early Cretaceous South American lizard †*Tijubina pontei* (figure 5*b*). We note the holotype specimen appears to be a juvenile based on unfused neural arches and centra (electronic supplementary material, figure S3).

*Entepicondylar and ectepicondylar foramen of humerus present.* The ectepicondylar foramen is nearly universally present in Lepidosauria, whereas the entepicondylar foramen is lost in squamates but retained in sphenodontians [6,7,64,73]. On the other hand, the absence of both foramina is considered a strong diagnostic trait for archosauromorphs [7,28,68,71,72], and it is one of the anatomical features supporting the sister group relationship between turtles and archosauromorphs [28]. Whiteside *et al*. [24] described the presence of both foramina in †*C. microlanius*; however, we were unable to observe any foramina in their only illustration (figure 1) of this feature. Additionally, our inspections of the CT scans did not reveal any detectable foramina on the entepicondyles or ectopicondyles (figure 4).

*Expanded radial condyle of the humerus absent* (*new observation*)*.* This feature was not discussed by Whiteside *et al*. [24], but it is one of the synapomorphies for squamates in the dataset used by that study. †*Cryptovaranoides microlanius* was scored as having the expanded radial condyle present in the phylogenetic dataset used by the authors, but our inspection of the CT scan data on both humeri of the holotype from different angles clearly shows this is absent in this taxon (figure 4).

*Ulnar patella absent* (*new observation*)*.* This feature was not discussed by Whiteside *et al*. [24] but it is one of the key features separating squamates from other reptiles (including other lepidosaurs) [7]. Although verification of the presence of patellae in fossils is difficult (see [74]) the good state of preservation of the forelimbs in †*C. microlanius* suggests the ulnar patella is absent in this taxon (figure 4).

### 3.3. The phylogenetic affinities of †*Cryptovaranoides microlanius* within reptiles

Dataset 1 was the matrix most suited for testing the broader affinities of †*Cryptovaranoides* within reptiles, as discussed above. In all of our results using dataset 1, †*Paliguana whitei* acted as a rogue taxon that contributed to poor resolution across the generated consensus topologies (electronic supplementary material, figures S3 and S4). Removing that species substantially improved the resolution (and most likely accuracy, [75]; figure 6; electronic supplementary material, figures S5–S7). All consensus trees place †*C. microlanius* outside Lepidosauria as either an archosauromorph or an indeterminate neodiapsid; the exact placement of †*C. microlanius* depends on the addition or removal of †*Paliguana* (electronic supplementary material, figures S3–S7)*.* Under the most robust phylogenetic hypothesis (without †*Paliguana*), †*C. microlanius* is inferred to be the sister to Allokotosauria within Archosauromorpha, albeit with weak support (figure 6; electronic supplementary material, figures S10–S12; see also list of synapomorphies supporting †*C. microlanius* within Archosauromorpha and its sub clades in the electronic supplementary material, Information).

The affinities of †*C. microlanius* to lepidosauromorphs are challenged by important reinterpretations of the postcranial skeleton of the holotype, including: absence of ectepicondylar and entepicondylar foramina (both originally described as present and critical to its placement within Lepidosauria; figure 4); absence of a radial condyle on the distal end of the humerus, which is commonly found in squamates and rarely elsewhere (figure 4); and absence of an ulnar epiphysis, which is unique to lepidosaurs (figure 4). Further, there is no evidence for a separate exit foramen for the vagus nerve in †*C. microlanius* (figure 1*d*; also acknowledged in Whiteside *et al*. [24]), thus making the presence of a divided metotic fissure unscorable in †*C. microlanius* (*contra* [24]). The opening for an anterior coracoid emargination identified by Whiteside *et al*. [24] is reinterpreted here as incomplete ossification of the coracoids, which is a common feature observed in juvenile reptiles, including ontogenetically immature fossils like the holotype of †*C. microlanius* (figure 5).

An archosauromorph identity for †*C. microlanius* is supported by the following characters: strong anterior emargination of the maxillary nasal process, which is rarely observed in squamates but is a hallmark feature of archosauromorphs, where it contributes to the formation of the antorbital fenestra (or fossa when the fenestra is absent; figure 1); and presence of an anterior process on the cervical ribs, which is uniquely found in archosauromorphs (incorrectly interpreted as a double-headed rib by [24]; figures 2 and 3).

**Figure 7.**
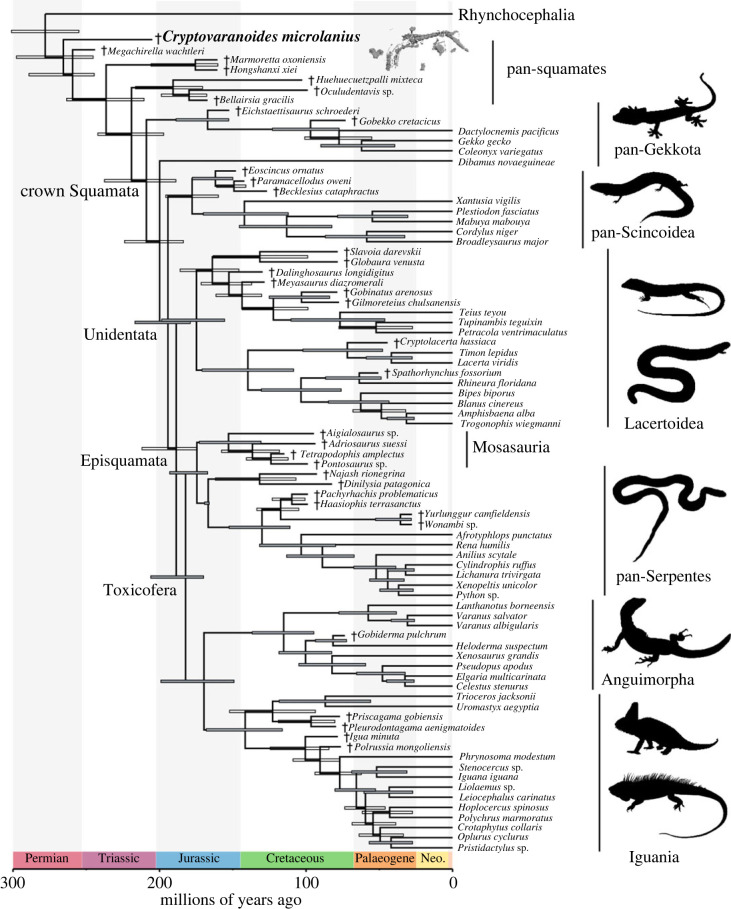
Phylogenetic relationships of †*Cryptovaranoides microlanius* (if constrained to lepidosaurs) using combined morphological and molecular data in dataset 2 (summarized tree). Maximum compatible tree (MCT) inferred using tip-dated Bayesian Inference analysis. Node bars indicate 95% highest posterior density intervals (HPDs), and grey bars indicate posterior support at nodes greater than 0.80. Daggers (†) indicate extinct species. For full trees, see the electronic supplementary material, figures S10–S17.

Datasets 2 and 3 are most suited to inferring relationships within lepidosaurs (see above). The analyses of datasets 2 and 3 provide strong evidence that the anatomy of †*C. microlanius* is incompatible with a crown squamate or anguimorph identity, even when this taxon is ‘forced’ to be a lepidosaur and tested using very different datasets. In analyses of dataset 2, †*C. microlanius* is inferred to be an early diverging pan-squamate (figure 7), well outside Anguimorpha or crown Squamata. That position is moderately supported by posterior probability (PP) values (0.7 and 0.83) and, in all these trees, †*C. microlanius* is subtended by extremely long branch lengths (electronic supplementary material, figures S12–S14). Using relaxed clock Bayesian inference, †*C. microlanius* is inferred to occupy a similar position but with lower support, less than 50% PP (electronic supplementary material, figure S15). These results suggest that, when †*C. microlanius* is included in a lepidosaur-specific dataset, the several character states it shares with some early lepidosauromorphs and archosauromorphs place it close to the root of the tree. We note that only four archosauromorphs are used as an outgroup in this dataset, and so it does not provide an adequate test of the non-lepidosaurian affinities of †*C. microlanius* (see results from dataset 1, figure 6).

**Figure 8.**
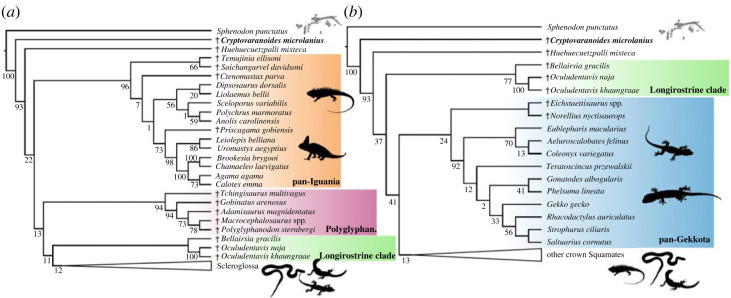
Testing the phylogenetic relationships of †*Cryptovaranoides microlanius* (if constrained to squamates) using morphological data in dataset 3. Strict consensus trees inferred with maximum parsimony from (*a*) the analysis of dataset 1 without and (*b*) with enforced constraints based on the consensus topology of living squamates from phylogenomic studies. For full trees, see the electronic supplementary material, figure S18.

Dataset 3 was analysed with and without the three species of stem-squamates added to the Gauthier *et al*. dataset in [10]: †*B. gracilis*, †*Oculudentavis khaungraae*, and †*Oculudentavis naja*. Undated parsimony and tip-dated Bayesian analyses failed to recover †*C. microlanius* within the squamate crown, again placing this species as a stem squamate, here one node stemward of the clade formed by †*B. gracilis*, †*Huehuecuetzpalli mixteca*, †*O. khaungraae*, and †*O. naja* (figure 8*a*,*b*).

### 3.4. Divergence times for reptiles and crown squamates

Divergence times among the major groups of reptiles are largely unaffected by the inclusion of †*C. microlanius* and its inferred placement within archosauromorphs (figure 6; electronic supplementary material, figure S13). For instance, in dataset 1, the median ages for Archosauromorpha, Crocopoda, and Allokotosauria all differ by less than approximately 1.3 Myr from previous estimates [28]. This result indicates the phylogenetic hypothesis provided here for †*C. microlanius* (as an archosauromorph) is largely compatible with the specimen age (contrary to its original phylogenetic hypothesis [24]). Furthermore, divergence times for the major clades of squamates using total evidence dating of dataset 2 (even when including †*C. microlanius*) are estimated to occur during the Middle and Late Jurassic (figure 7; electronic supplementary material, figure S21), in agreement with previous estimates [5,7,9,13–15,26]. The revised, more basal position of †*C. microlanius* is again more consistent with its age.

## 4. Discussion

The Late Triassic reptile †*C. microlanius* was originally interpreted as nested within Anguimorpha [24], which is a clade deeply nested within crown squamates [1,3,5–7,12–14,25,76–78]. If this interpretation of †*C. microlanius* is accurate, it would radically alter all previous hypotheses on the timing of squamate diversification, and potentially suggest widespread bias towards younger age estimates for squamates and other reptiles in timetrees produced using a wide variety of methods and both morphological and molecular data [1,5,7,9,13,14,24,25,78]. Specifically, the anguimorph affinity of †*C. microlanius* posited by Whiteside *et al*. [24] would suggest that several major components of squamate diversity would be tens of millions years older than previously thought.

Reinterpretation of the original data and analyses strongly reject a crown squamate identity for †*C. microlanius*. First, we find no evidence for referring most of the other specimens noted by Whiteside *et al*. [24] to this species. Secondly, new anatomical evidence obtained from the CT scan data indicates that several features used to link †*C. microlanius* to squamates and anguimorphs are in fact not observable (e.g. not preserved), poorly preserved and of ambiguous interpretation, or incorrectly described (see the electronic supplementary material). We provide a thorough redescription of the holotype and provide, for the first time to our knowledge, detailed images of key anatomical traits that highlight several traits seen in †*C. microlanius* that are incompatible with a lepidosaur hypothesis, and instead support its affinity to archosauromorphs. Finally, phylogenetic analyses of three separate datasets with radically different taxonomic composition and criteria of character construction, under multiple optimality criteria, consistently reject the hypothesis that †*C. microlanius* is a crown squamate. Instead, our analyses find that †*C. microlanius* is a neodiapsid of unclear placement with potential affinities to early archosauromorphs.

Crown reptiles (turtles, archosauromorphs and lepidosauromorphs) underwent extensive radiation and diversification during the Early to Middle Triassic but have roots dating back into the Permian [23]. While numerous archosauromorph subclades are known to have converged on similar body plans [71,79–84], the same level of scrutiny has not been extended to a number of lepidosaur-like anatomies and morphologies observed among archosauromorphs.

For example, †*C. microlanius* shares with crown squamates an elongated (rod-like) squamosal and a large coronoid bone with a prominent dorsal process. Protorosaurian archosauromorphs [7,28,48,82] possess several anatomical features shared with squamates, including the absence of a complete lower temporal bar (also occurring in Mesozoic marine reptiles and numerous other neodiapsids), a posteriorly emarginated quadrate bone and quadrate conch (also occurring in Mesozoic marine reptiles), a hooked fifth metatarsal, and occasionally, pleurodont dentition (e.g. in kuehneosaurids) [27,48,82,85–88]. Squamate-like features are also found in rhynchosaurs and allokotosaurs, such as a hooked fifth metatarsal and a well-developed coronoid bone on the mandible [27,28,48,72,86,87] (the latter being present and the former unknown in †*C. microlanius*). Even when considering highly derived members of the archosauromorph tree, we can find features of interest that have been historically linked to lepidosaurs in systematic studies. For instance, hatchlings of *Alligator mississippiensis* have their first tooth generation not attached in a socket made of alveolar bone, but rather to the labial face of the medial wall of the tooth bearing element; that is, hatchling *A. mississippiensis* are (at least partially) pleurodont [89]. Although it is not currently known how widespread this condition is across crocodilians and in other archosaurs, it is possible that it is a juvenile feature of thecodont neodiapsids, and that pleurodont lepidosauromorphs retain the juvenile state of their saurian sister-groups [89]. Further, the practice of using discrete tooth implantation modes as characters is problematic; tooth implantation types are difficult to distinguish even using histological sections [89,90]. Therefore, lepidosauromorph (and more specifically, squamate) features traditionally considered to be characteristic or even unique to these groups [3,6] are now recognized to be widespread across other groups of crown reptiles, including several groups of archosauromorphs, including †*C. microlanius*.

Recently, Triassic formations in England have produced several diapsids that may be early diverging lepidosaurs, as well as plentiful examples of early rhynchocephalians [20–22,24,91,92]. One of these, †*Feralisaurus corami*, was described from a relatively complete but heavily crushed anterior skeleton from the Middle Triassic Otter Sandstone Formation and was suggested to be a stem lepidosaur [92]. Although †*C. microlanius* and †*F.corami* are generally similar, the former possesses a proportionately longer neck and lacks fenestration of the coracoids (see the electronic supplementary material). As such, †*C. microlanius* and †*F.corami* are not likely to be conspecific; however, presence of two broadly similar taxa highlights the need for caution when referring isolated elements.

Our re-evaluation of the phylogenetic affinities of †*C. microlanius* has larger implications for the interpretation of lepidosaur-like skeletons from the Triassic reptile assemblages of the UK and other Triassic faunas—see further discussion on the difficult interpretation on other Triassic fissure fill deposits previously linked to lepidosaurians in ([7,9] electronic supplementary material). Our analyses clearly show that †*C. microlanius* is neither an anguimorph nor a crown squamate, and perhaps not a member of any crown lepidosaur or archosaur clade. We urge a critical approach using different methodologies and datasets to assess the relationships of squamate- and lepidosaur-like reptiles from the Triassic. In spite of the production of large datasets sufficient to broadly test reptile relationships [7,28,82,93], the bedrock of phylogenetic analysis using morphological characters is the thorough assessment of character presence, absence and homology. We also note that the construction of chimaeric hypodigms remains a problem in palaeontology (for example, [94]) that has deleterious effects on higher order hypotheses, such as phylogenies and subsequent studies of patterns and processes of evolution and the timing of clade origins.

Although the phylogenetic affinities of †*C. microlanius* remain relatively unclear, this species shares several features with archosauromorphs, the total clade of birds and crocodilians [7,48,71–73,95,96]. †*Cryptovaranoides microlanius* is placed as the sister of Allokotosauria, a diverse Triassic archosauromorph clade outside the crown group, with moderate support in analyses of dataset 1 that exclude †*Paliguana*. However, several anatomical features of †*C. microlanius*, especially the morphology of its dentition, markedly differ from the conditions observed in allokotosaurs. It appears that †*C. microlanius* is part of a poorly known radiation of early small-bodied archosauromorphs, but the phylogenetic affinities of this species will only be resolved by future fossil discoveries. †*Cryptovaranoides* highlights a potential new branch in the exceptional Triassic radiation of crown reptiles and demonstrates the probability that key small-bodied clades might still await discovery (also see [84]).

## Ethics

This work did not require ethical approval from a human subject or animal welfare committee.

## Data Availability

All data needed to evaluate the conclusions in the paper are present in Dryad Digital Repository: https://doi.org/10.5061/dryad.8kprr4xtn [97], the paper and/or the electronic supplementary material [98].
